# Carbonic anhydrase inhibitors improves cognitive functions in Tg2576 AD mice

**DOI:** 10.1002/alz.092822

**Published:** 2025-01-03

**Authors:** Rafael Vazquez‐Torres, Rebecca M Parodi‐Rullan, Ashley M Carey, Silvia Fossati

**Affiliations:** ^1^ Alzheimer’s Center Lewis Katz School of Medicine, Temple University, Philadelphia, PA USA; ^2^ Lewis Katz School of Medicine, Temple University, Philadelphia, PA USA; ^3^ Alzheimer’s Center at Lewis Katz School of Medicine, Temple University, Philadelphia, PA USA

## Abstract

**Background:**

Over the years, Alzheimer’s Disease (AD) has been identified as a multifactorial disease, with cerebral vascular dysfunction being one of the most common and early pathological features. Vascular risk factors (VRF) are thought to further increase AD risk and pathology. Cerebral Amyloid Angiopathy (CAA) is defined as the accumulation of amyloid‐beta (Aβ) on the vascular wall. CAA occurs progressively with age in human AD brains, vascular dementias, and the Tg2576 AD mouse model. The Tg2576 develops significant amounts of parenchymal Aβ plaques and vascular amyloid deposits making it a valuable model to study CAA and AD. To study the contribution of VRF to CAA and AD pathology, we induced hyperhomocysteinemia (HHcy) and hypertension (HP) in Tg2576 mice. We have shown that carbonic anhydrase inhibitors (CAi) decreased vascular amyloid accumulation and neuroinflammation and prevented cognitive impairment in TgSwDI mice. Here, we evaluated the effects of HP and HHcy in AD and CAA pathology. In addition, we assessed the beneficial effects of CAi in the Tg2576 with/without the presence of VRF.

**Methods:**

To induce VRF, male and female WT and Tg2576 mice were fed a HHcy inducing diet and/or were given L‐NAME (NO inhibitor to induce HP) in the water starting at 5 months of age. To test the effects of CAi, the FDA‐approved CAi (Acetazolamide) was added to the diet starting at 5 months of age. Animals were then tested in a battery of behavioral measures including spatial learning and memory (barnes maze, fear conditioning, novel object recognition, open field, and rotarod), at 12 months of age.

**Result:**

The presence of HHcy or HT worsens spatial learning and memory in the WT and Tg2576 mice. Preliminary results may suggest that treatment with CAi improved spatial learning and memory in the WT and Tg2576 mice, in the presence or absence of VRFs.

**Conclusion:**

Taken together, our results suggest that VRF affect learning and memory in AD/CAA and non‐AD/CAA animal models. Importantly, we demonstrate that CAi is a promising treatment for treating mixed AD and CAA pathology.

